# Autosomal dominant tubulointerstitial kidney disease (ADTKD) in Ireland

**DOI:** 10.1080/0886022X.2019.1655452

**Published:** 2019-09-11

**Authors:** S. Cormican, D. M. Connaughton, C. Kennedy, S. Murray, M. Živná, S. Kmoch, N. K. Fennelly, P. O’Kelly, K. A. Benson, E. T. Conlon, G. Cavalleri, C. Foley, B. Doyle, A. Dorman, M. A. Little, P. Lavin, K. Kidd, A. J. Bleyer, P. J. Conlon

**Affiliations:** a Nephrology Department, Beaumont Hospital, Dublin, Ireland;; b Department of Medicine, Boston Children’s Hospital, Harvard Medical School, Boston, MA, USA;; c Trinity Health Kidney Centre, Trinity Translational Medicine Institute, Dublin, Ireland;; d Department of Medicine, Royal College of Surgeons, Dublin, Ireland;; e Department of Pediatrics and Adolescent Medicine, Research Unit for Rare Diseases, First Faculty of Medicine, Charles University, Prague, Czech Republic;; f Pathology Department, Beaumont Hospital, Dublin, Ireland;; g Clinical Research Centre, Royal College of Surgeons, Dublin, Ireland;; h Trinity Health Kidney Centre, Tallaght Hospital, Dublin, Ireland;; i Section on Nephrology, Wake Forest School of Medicine, Winston-Salem, NC, USA

**Keywords:** ADTKD, genetic, MUC-1, UMOD, chronic kidney disease, HNF-1B, urinary smear, frameshift

## Abstract

**Introduction:** Autosomal dominant tubulointerstitial kidney disease (ADTKD) is a rare genetic cause of renal impairment resulting from mutations in the *MUC1, UMOD, HNF1B, REN*, and *SEC61A1* genes. Neither the national or global prevalence of these diseases has been determined. We aimed to establish a database of patients with ADTKD in Ireland and report the clinical and genetic characteristics of these families.

**Methods:** We identified patients via the Irish Kidney Gene Project and referral to the national renal genetics clinic in Beaumont Hospital who met the clinical criteria for ADTKD (chronic kidney disease, bland urinary sediment, and autosomal dominant inheritance). Eligible patients were then invited to undergo genetic testing by a variety of methods including panel-based testing, whole exome sequencing and, in five families who met the criteria for diagnosis of ADTKD but were negative for causal genetic mutations, we analyzed urinary cell smears for the presence of MUC1fs protein.

**Results:** We studied 54 individuals from 16 families. We identified mutations in the *MUC1* gene in three families, *UMOD* in five families, *HNF1beta* in two families, and the presence of abnormal MUC1 protein in urine smears in three families (one of which was previously known to carry the genetic mutation). We were unable to identify a mutation in 4 families (3 of whom also tested negative for urinary MUC1fs).

**Conclusions:** There are 4443 people with ESRD in Ireland, 24 of whom are members of the cohort described herein. We observe that ADTKD represents at least 0.54% of Irish ESRD patients.

## Introduction

Autosomal dominant tubulointerstitial kidney disease (ADTKD) is a rare genetic cause of progressive chronic kidney disease (CKD) and end-stage renal disease (ESRD) [1]. Causative mutations have been identified in the *MUC1* gene encoding mucin-1, the *UMOD* gene encoding uromodulin (previously Tamm-Horsfall protein), the *REN* gene encoding renin, the *HNF1B* gene encoding hepatocyte nuclear factor 1beta, and the *SEC61A1* gene encoding translocon subunit SEC61A [1–4]. The cardinal features include gradual loss of kidney function, histological evidence of tubular atrophy and interstitial fibrosis and an autosomal dominant inheritance pattern. Urinary sediment is typically bland. The mean age of ESRD is approximately 45 years but this is variable [5,6]. KDIGO consensus guidelines recommend criteria for diagnosis of confirmed or suspected ADTKD [7].

Genetic testing for *UMOD, REN,* and *HNF1B* mutations is reasonably straightforward. For ADTKD-*MUC1*, identification of causative mutations was recalcitrant to whole exome and whole genome sequencing and ultimately required cloning, standard Sanger sequencing and *de novo* assembly of *MUC1* genomic sequence. The identified mutations were cytosine duplications in one of the heptanucleotide cytosine tracts that is present in each of the 20–125 canonical 60 base pair variable number of tandem repeat (VNTR) units of *MUC1* [8]. Duplication of a cytosine results in the mistake, a frameshift, in protein translation and the production of a unique and structurally abnormal mucin protein, MUC1fs (MUC1 frameshift protein). It is thought that MUC1fs deposition results in accelerated apoptosis of renal tubular cells, tubulo-interstitial fibrosis, and progressive chronic kidney disease [1]. Other mutations (e.g., an adenosine insertion, guanosine insertion, deletion of two cytosine residues, or duplication of 16 base pairs) causing the same frameshift can also cause ADTKD-*MUC1* [9,10]. Due to the high guanosine-cytosine content and the number of repeats, the *MUC1* gene cannot be sequenced by traditional methods.

At present, genetic analysis for the most prevalent mutations 27dupC and 28dupA can be obtained by the Clinical Laboratory Improvement Amendments of 1988 (CLIA) certified mass-spectrometry-based genotyping assay provided at the Broad Institute [10,11]. Identification of other unique frameshift mutations is possible by Illumina sequencing and specific bioinformatic analysis of the VNTR region of *MUC1* [10]. However, not all mutations resulting in ADTKD-*MUC1* can be determined in this manner [12,13]. A recent development in the diagnosis of ADTKD-*MUC1* is the detection of abnormal MUC1fs protein in urine samples from individuals with suspected ADTKD. This technique relies on immunohistochemical intracellular detection of the unique disease-causing frameshift protein on urinary cell smears [10].

In Ireland, 11.8% of the population has CKD [14] and more than 4400 people, 928 per million population, have ESRD [15]. Genetic causes of CKD e.g., Autosomal dominant polycystic kidney disease and Alport syndrome have been described in the Irish population previously [16]. Recently, the Irish Kidney Gene Project (IKGP) indicated that 34% of patients attending Irish nephrology units have a relevant family history [17]. Patients with unspecified tubulointerstitial kidney disease were significantly more likely to have a family history of kidney disease than those with other causes of CKD. Whole exome sequencing of 114 Irish families with CKD identified a monogenic cause of CKD in 37% [18]. The IKGP and subsequent work enabled identification of individuals in Ireland who might suffer from ADTKD.

We set out to identify individuals recruited through the IKGP who had clinical characteristics, family history, and renal biopsy results (where available) in keeping with ADTKD and to identify causative mutations in these patients. Of 1840 individuals included in the IKGP study, 29 had a history of unspecified tubulointerstitial kidney disease and two-thirds of these reported a relevant family history [17].

The goals of this work were to identify a cohort of Irish patients with ADTKD and to describe clinical characteristics and causative mutations in our population. We aimed to provide an estimate of prevalence within the Irish population. Furthermore, establishing a database of Irish patients with ADTKD will allow us to contribute to global progress in the understanding of this disease. This could include future enrollment of Irish patients with ADTKD in clinical trials of disease modifying agents.

## Methods

The IKGP was a multi-centre cross-sectional study of adult patients with CKD attending Irish nephrology units. Ethical approval for patient enrollment, collection of clinical data, and DNA collection was granted by each local medical ethics committee. Patients provided written consent at enrollment. The full IKGP study protocol has been described elsewhere [17].

Nineteen individuals were identified via the IKGP study as meeting criteria for suspected ADTKD. An additional 23 relatives were recruited to give a total of 42 individuals from 11 families. Patients referred to the Renal Genetics Clinic in Beaumont Hospital were also considered, yielding a further 12 individuals from five families.

We then performed a review of electronic medical records, biochemical and histologic (kidney biopsy) results to compile a database of clinical characteristics. Some data fields (e.g., proteinuria quantification at diagnosis) were not available for all patients and in these cases was recorded as missing.

We included only patients who met KDIGO diagnostic criteria for a definite ADTKD diagnosis (See Table 1). Inclusion criteria were as follows: (1) A clinical history compatible with ADTKD and either (2) A genetic diagnosis in the patient or affected family member OR (3) A family history suggestive of autosomal dominant inheritance of kidney disease AND a kidney biopsy compatible with a diagnosis of ADTKD in the patient or an affected family member. Exclusion criteria were as follows: (1) Clinical characteristics suggestive of an alternative diagnosis (including hematuria, drug exposure accounting for renal pathology, suggestion of an alternative diagnosis on renal biopsy); (2) Non-availability of samples for genetic testing in at least one member of an affected family.

**Table 1.** KDIGO criteria for suspected and definite diagnosis of ADTKD which were used to define inclusion in our study.

Suspected diagnosis	Confirmed diagnosis
Family history of autosomal dominant inheritance of CKD with clinical characteristics in keeping with ADTKD	Compatible family history (minimum of 1 first degree relative) and compatible kidney biopsy in one affected individual
Without a compatible family history – compatible histology on a kidney biopsy of compatible extra-renal mutations e.g., Gout or Diabetes	Demonstrated mutation in one of the 4 known genes (*MUC1, UMOD, HNF1B*, and *REN*)

ADTKD: autosomal dominant tubulointerstitial kidney disease; CKD: chronic kidney disease.

Collection and bio-banking of DNA samples was carried out by a Rare Kidney Disease Registry and Biobank in Trinity Kidney Health Centre and the Royal College of Surgeons in Ireland.

We initially performed targeted genetic testing for pathogenic mutations in the *MUC1* and *UMOD* genes (facilitated through Anthony Bleyer, Wake Forest School of Medicine, Winston-Salem, NC USA or the Broad Institute). Genetic testing for *MUC1* mutations was carried out at the Broad Institute of Harvard University and the Massachusetts Institute of Technology, Cambridge, MA as previously described [11]. This analysis identified individuals with previously described cytosine duplication (27dupC) or adenine duplication (28dupA) in the VNTR of the *MUC1* gene [10]. Genetic testing for *UMOD* mutations was carried out by the Rare Inherited Kidney Disease Team, Wake Forest School of Medicine, Winston-Salem, USA using full sequence analysis of the *UMOD* gene [19].

**Figure 1.**
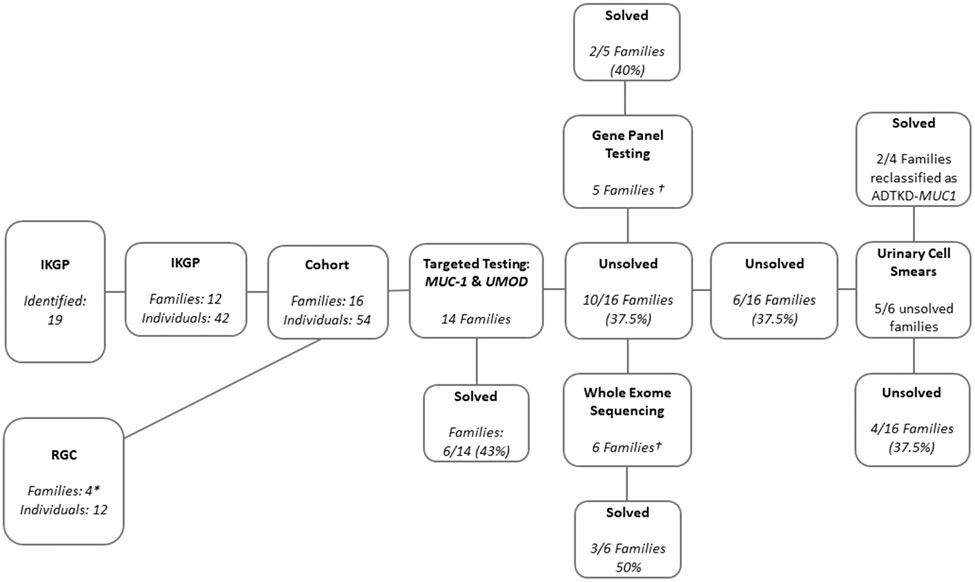
Flow chart demonstrating recruitment to this cohort and methods of genetic testing for ADTKD. Sixteen families have been identified with a total solve rate of 12/16. Individuals with tubulointerstitial kidney disease and a relevant family history identified from the Irish Kidney Gene Project (IKGP) were further screened via review of biopsy reports and clinical records. Five individuals were excluded from this report because they did not meet KDIGO criteria for a definite diagnosis of ADTKD, 3 because they did not have an affected first degree relative (or confirmed mutation) and 2 because they did not have a compatible biopsy report (or confirmed mutation). The Renal Genetics Clinic (RGC) was an additional source of recruitment. *Two families recruited via the RGC went directly to gene panel testing without prior targeted testing for MUC1 and UMOD mutations. †One individual had both gene panel and whole exome sequencing and was found to have a pathogenic mutation in the UMOD gene on both methods.

Families without an identified pathogenic mutation in either *UMOD* or *MUC1* underwent genetic testing by either whole exome sequencing (SeqCap EZ Exome v3.0) or gene panel testing (individuals from five families) on a custom gene panel of 227 genes (Supplementary Table 1). Figure 1 shows the sequential use of genetic testing strategies. Whole exome sequencing was performed in Boston Children’s Hospital as previously described [18,20].

Gene panel testing was performed in the Royal College of Surgeon’s Ireland. Library preparation was conducted using standard library preparation methods (Roche Diagnostics). Sequencing was conducted using Illumina MiSeq and NextSeq sequencers. Sequencing data were analyzed using an in-house bioinformatic pipeline. Sequencing reads were aligned to the reference genome using Burrows-Wheeler Aligner [21], and the Genome Analysis Toolkit (GATK) pipeline [22] was used for base quality score recalibration, indel realignment, and duplicate removal. Variant identification was conducted using GATK standard hard filtering parameters [23]. ANNOVAR [24] was used for variant annotation, and variants were assessed for pathogenicity following guidelines of the American College of Medical Genetics [25].

As discussed above, the MUC1fs protein can be detected by immunohistochemical staining of urinary cell smears. In six individuals without a previously identified genetic diagnosis, we collected urinary samples for immunohistochemical analysis to identify MUC1fs protein. This was performed by Dr. Živná in Research Unit for Rare Diseases in the First Faculty of Medicine, Charles University, Prague, as previously described [10].

Following the above diagnostic methods, 5 individuals remained in the ADTKD-NOS group.

In accordance with the 2015 KDIGO guidelines (2015), patients were categorized as ADTKD-*MUC1*, ADTKD-*UMOD, ADTKD-HNF1ß*, or ADTKD-NOS. Diagnostic criteria are shown in Table 1.

Descriptive statistical analysis and statistical testing for differences between were performed using Minitab 18^®^ (Minitab, State College, PA) statistical software. Differences in mean values between groups were compared using one-way ANOVA testing and differences in proportion by group were compared using Chi-Square testing. Differences in mean values for those with and without an identified mutation were compared using two-sample *t*-testing. A *p* value of <0.05 was deemed statistically significant.

## Results

### Overall cohort

**Figure 2.**
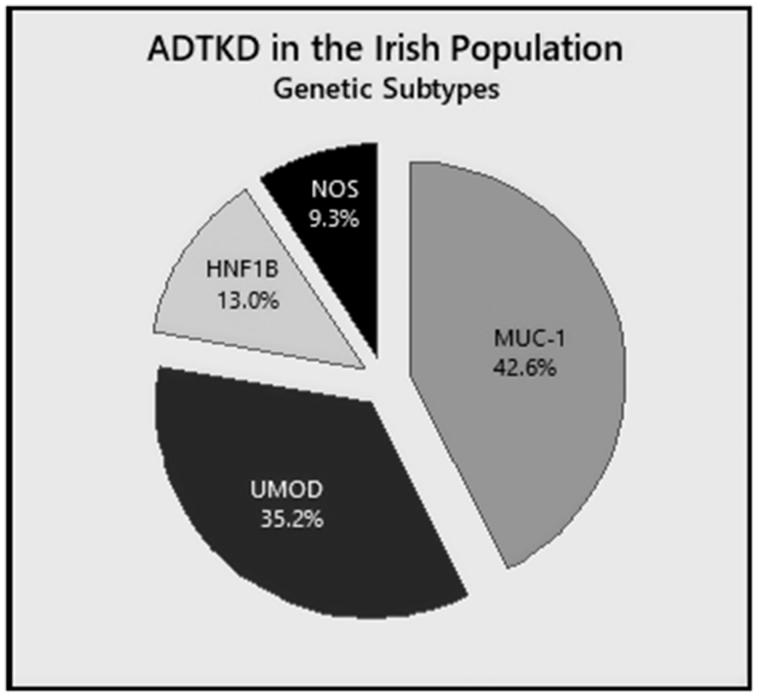
Frequency of each genetic subtype of ADTKD within this Irish cohort. MUC1: ADTKD-MUC1; UMOD: ADTKD-UMOD; HNF1B: ADTKD-HNF1B; NOS: not otherwise specified.

This case series describes 16 families comprised of 54 individuals that meet KDIGO guideline definitions for ‘Definite ADTKD’. Five families have ADTKD-*MUC1* (in two of which diagnosis was based on urinary intracellular detection of MUC1fs), five ADTKD-*UMOD*, and two ADTKD-*HNF1B* (Figure 2). Following the above diagnostic methods, five individuals (from four families) remained in the ADTKD-NOS group. Although we were unable to diagnose a pathogenic mutation, these individuals had both a compatible biopsy (in at least one family member) and an autosomal dominant family history of kidney disease. Therefore, they meet criteria for confirmed ADTKD by KDIGO guidelines [7].

**Figure 3.**
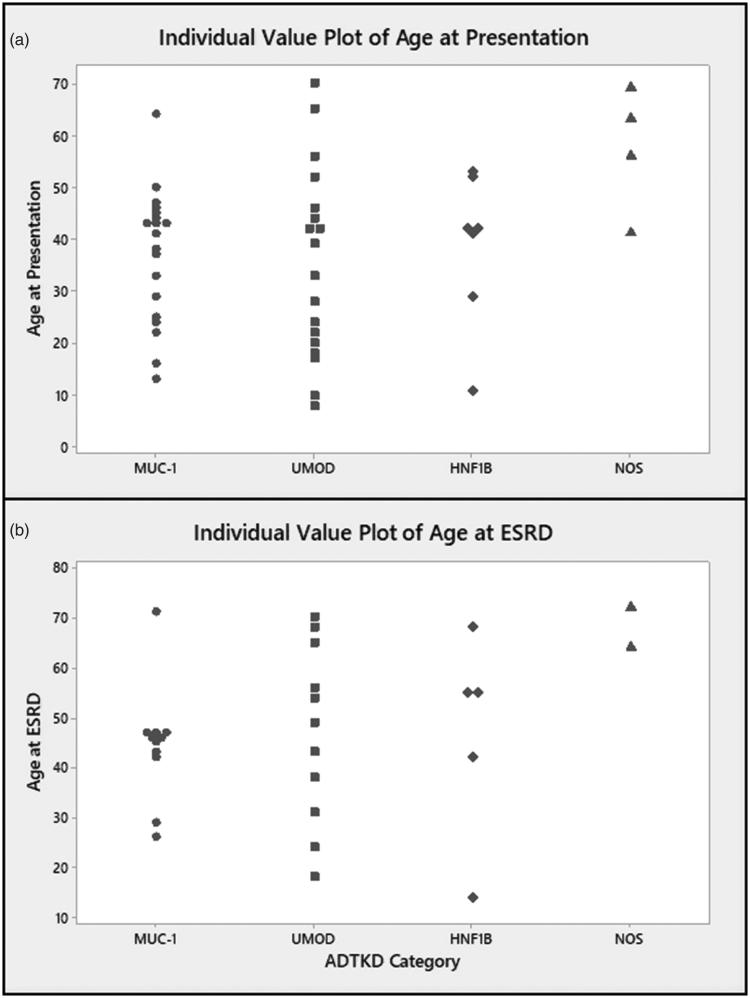
(a) Age at presentation and (b) Age at end stage renal disease (ESRD) by genetic category of ADTKD. MUC1: ADTKD-MUC1; UMOD: ADTKD-UMOD; HNF1B: ADTKD-HNF1B; NOS: not otherwise specified.

In the overall cohort 28/54 (52%) individuals were male, the mean age at presentation is 38.3 ± 16.0 years and the mean creatinine at presentation is 210.6 ± 163.5 micromoles/litre (µmol/L). ESRD was reached by 32/54 individuals (57%) at a mean age of 47.4 years (Table 2). Figure 3 demonstrates the range of ages at presentation (a) and ESRD (b) by genetic category.

**Table 3.** Each family included in this cohort showing genetic mutation identified. Details of numbers of affected individuals with confirmed mutation and compatible biopsy are shown. The number of 1st degree relatives affected is shown for the ADTKD-NOS families.

Family number	ADTKD category	Genetic mutation	Protein	Testing laboratory	Number individuals in family	Number with confirmed mutation	Number with compatible renal biopsy	Number 1st degree relatives (ADTKD-NOS)
**1**	*MUC1*	Insertion in VNTR region	frameshift	BI	13	7	5	
**2**	*MUC1*	Insertion in VNTR region	frameshift	BI	4	2	0	
**3**	*MUC1*	Insertion in VNTR region	frameshift	BI	2	1	1	
**4**	MUC1*	NA	frameshift	CU	3	0	2	
**5**	MUC1*	NA	frameshift	CU	1	0	1	
**6**	*UMOD*	c.821A > G	p.Y274C	BG	2	2	1	
**7**	*UMOD*	c.668G > A	p.C223Y	BG	5	3	4	
**8**	*UMOD*	c.317G > A	p.C106Y	BG	7	2	1	
**9**	*UMOD*	c.1382C > A	p.A461E	BCH	4	1	1	
**10**	*UMOD*	c.280T > C	p.C94R	BCH/RCSI**	1	1	0	
**11**	*HNF1B*	c.1333_1334delGC	p.Ala445fs*105	BCH	4	4	2	
**12**	*HNF1B*	c.544 + 3_544 + 6	Del 75% ESS	BCH	3	3	0	
**13**	NOS	NA	NA	NA	2	0	1	2
**14**	NOS	NA	NA	NA	1	0	1	1
**15**	NOS	NA	NA	NA	1	0	1	2
**16**	NOS	NA	NA	NA	1	0	1	2

ADTKD: autosomal dominant tubulointerstitial kidney disease; BCH: Boston Children’s Hospital; BI: broad institute; BG: Bowman Grey Institute; CU: Charles University, Prague; del: deletion; ESS: essential splice site; fs: frameshift; NA: non-applicable; RCSI: Royal College of Surgeons Ireland (gene panel analysis); VUS: variant of uncertain significance. *Urinary MUC1fs protein detected, no detected genetic mutation to date; **Mutation identified in both centres.

Twenty-four individuals included in this report are currently living with ESRD. The most recent national renal office figures indicate that 4443 patients are living with ESRD in Ireland [15]. We therefore estimate that ADTKD accounts for at least 0.54% of ESRD cases in Ireland.

### Identification and clinical characteristics of individuals with ADTKD-MUC1

**Figure 4.**
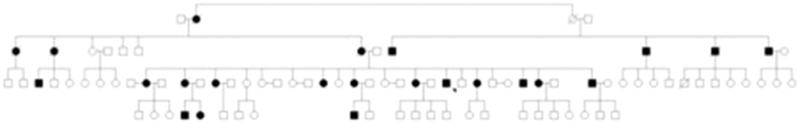
Family tree demonstrating a large Irish family with cases of ADTKD over four generations due to MUC1 genetic mutations. Shaded = Affected; Unshaded = Unaffected; Box = Male; Circle = Female; Strikethrough = Deceased.

This group included families with a confirmed *MUC1* frameshift mutation and families with mutant MUC1fs protein identified on urinary cell smears. This category includes 5/16 families (23%) and is comprised of 23 individuals. In 12/23 (52%) individuals, we identified a pathogenic mutation in the *MUC1* gene and seven others were categorized as ADTKD-*MUC1* based on clinical characteristics and detection of a pathogenic in *MUC1* in a relative. In each of these cases, a frame shift mutation (either a 27dupC or 28dupA in the VNTR region) in the *MUC1* gene was identified. A pedigree of the largest family affected by ADTKD-*MUC1* is shown in Figure 4.

We identified MUC1fs protein in urinary cell smears from three individuals (two from the same family and in one other individual). These families were not included in the previous report of this technique [10]. We reclassified these individuals and one other affected first degree relative as ADTKD-*MUC1*. To date we have been unable to identify a pathogenic mutation on genomic sequencing of the *MUC1* gene. Age at presentation in these four patients was slightly older (42.5 years).

In the ADTKD-*MUC1* group, 8/23 (35%) individuals were male. These 23 individuals presented at a mean of 37 years and with a mean serum creatinine of 136 µmol/L. Twelve of 23 (52%) patients have reached ESRD at a mean age of 42 years.

### Identification and clinical characteristics of individuals with ADTKD-UMOD

**Figure 5.**
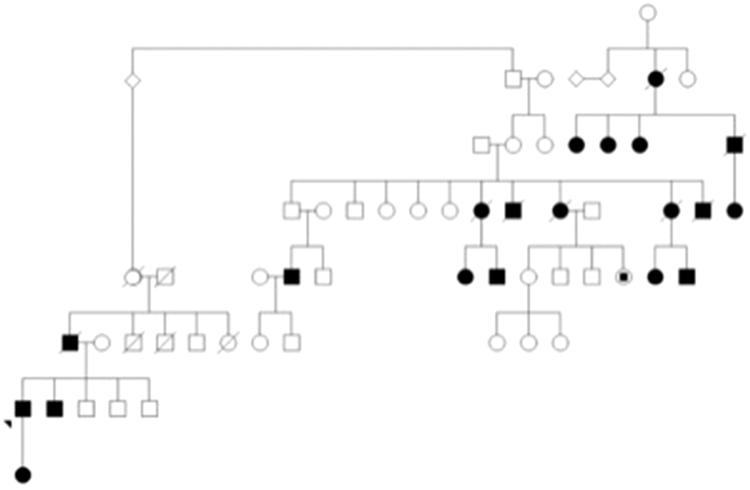
Family tree demonstrating a large Irish family with cases of ADTKD over four generations due to UMOD mutations. Shaded = Affected; Unshaded = Unaffected; Square within a circle = Status Unknown; Box = Male; Circle = Female; Strikethrough = Deceased.

This category includes 5/16 (31%) families and is comprised of 19 individuals. In 9/19 (47%) individuals we identified a pathogenic mutation in the *UMOD* gene. The remaining individuals were categorized as ADTKD-*UMOD* based on clinical characteristics and detected pathogenic gene mutation in a relative. Details of identified mutations are given in Table 3 and have been previously reported [18,26–28]. Of note, three of the families included here were previously reported (Families 8–10 in Table 3 or B2337, P193, and P232 in the report by Connaughton et al. [18]. The others had been previously identified by testing in the Wake Forest School of Medicine. Eighty percent of families had a history of gout (4/5). A pedigree of the largest family affected by ADTKD-*UMOD* is shown in Figure 5.

**Table 2.** Clinical characteristics of individuals in this cohort by ADTKD-subgroup.

	ADTKD (All) *n* = 54	ADTKD-*MUC1n* = 23	ADTKD-*UMOD* n = 19	ADTKD-*HNF1B* n = 7	ADTKD-NOS *n* = 5	*p* Value
Confirmed mutation *n* (%)	28 (52%)	15*/23 (65%)	9 (48%)	7 (100%)	0 (0%)	NA
Number of families	16	5	5	2	4	NA
Mean age at presentation, years (SD)	38 (16.0)	37.0 (12.8)	35.3 (18.3)	38.6 (14.5)	57.25 (12.0)	0.090**
Mean creatinine at presentation, µmol/l (SD)	210.6 (163.5)	136.4 (45.6)	272 (217.2)	171 (20.4)	372 (214.0)	0.030**
Number reached ESRD (%)	32 (59%)	12 (52%)	12 (63%)	5 (71%)	3 (56%)	NA
Mean age at ESRD, years	47.4 ( 16.0)	44.5 (11.47)	46.9 (17.8)	46.8 (20.5)	68.0 (5.7)	0.303**
Gout (%)	15/54 (27%)	0% (0/19)	68% (13/19)	14% (1/7)	20% (1/5)	<0.001***

ADTKD: autosomal dominant tubulointerstitial kidney disease; ESRD: end stage renal disease; *n*: Number; NA: non-applicable; SD: standard deviation. *For two individuals in this group, diagnosis was based on urinary detection of MUC1fs protein in urinary smears; **p Values by one-way ANOVA testing comparing ADTKD sub-categories; ***p Values by chi-square testing comparing ADTKD sub-categories.

In the ADTKD-*UMOD* group, 13/19 (68%) individuals were male. These 19 individuals presented at a mean age of 35.3 years and with a mean serum creatinine of 272 µmol/L. In this group, 12/19 (63%) patients have reached ESRD at a mean age of 46.9 years.

### Identification and clinical characteristics of individuals with ADTIKD-HNF1B

This category includes 2/16 (12.5%) families and is comprised of seven individuals. All seven individuals have had genetic testing confirming a pathogenic mutation in the *HNF1B* gene. These families were previously included in a report by Connaughton et al. [18].

In the ADTKD-*HNF1B* group, 3/7 (43%) individuals were male. These seven patients presented at a mean age of 39 years with a mean serum creatinine of 171 µmol/L. Five of these patients (71%) progressed to ESRD at a mean age of 47 years. Patients with pathogenic mutation in *HNF1-B* also had concomitant conditions including diabetes (*n* = 2) and abnormal LFTs (*n* = 2). Hypomagnesemia was not ascertained in this cohort. Hyperuricemia or gout occurred in 4/7 individuals (57%). One individual (from family 11) had a renal ultrasound showing renal cysts in the native kidneys.

Of note, both of these families (Family 11 & 12 in Table 3) have been reported previously (B2342 and P306 in the report by Connaughton et al. [18]). One member of family 12 had had prior unilateral nephrectomy due to vesicoureteric reflux and was previously classified as CAKUT (Congenital Anomalies of the Kidney and Urinary Tract). Given that genetic testing led to a reclassification of the diagnosis we have included that family in this report.

### Identification and clinical characteristics of individuals with ADTKD-NOS

This category includes 4/16 (37.5%) of families and is comprised of five individuals who meet KDIGO criteria for a definite diagnosis of ADTKD based on family history and renal biopsy findings in whom we have not yet identified a causative mutation. This is despite gene panel testing for 227 genes, including *UMOD*, *MUC1*, *HNF1B* and *REN* mutations and examination or urine smears for mutant MUC1fs (in 3/4 families). These families were small with only one available for testing in all but one case and so did not lend themselves to linkage studies.

In this group, 4/5 (80%) of individuals were male. The mean age at presentation in this group was 57 years, compared to 37 in those individuals with an identified mutation (*p* = 0.049). The mean age of the five patients in this group, who progressed to ESRD was 68 years, which was older than a mean of 46 years for individuals with an identified mutation (*p* = 0.047).

## Discussion

ADTKD is a relatively recently described clinical entity and clinical knowledge of the disease is growing [2,5,29]. This is the first published report of ADTKD in Ireland. Pathogenic mutations in the *MUC1*, *UMOD*, and *HNF1B* genes have been identified in a 10 Irish families to date and a further two families have had MUC1fs protein identified in urinary cell smears.

Knowledge regarding the prevalence of ADTKD is limited. A previous Austrian study estimated that ADTKD due to *UMOD* mutations accounted for 0.073% of cases of ESRD in Austria [30]. A Spanish study recently reported 131 individuals from 56 families affected by ADTKD [31] but did not comment on how this related to national prevalence. In the UK, a survey of nephrology patients attending a tertiary referral centre estimated prevalence of 9 cases per million of ADTKD-*UMOD* and 17.5 cases per million of all causes ADTKD – based on family history and genotyping for *UMOD* mutations [32]. This study estimated that ADTKD-*UMOD* alone accounts for 2% of cases of ESRD.

We sought to estimate the frequency of ADTKD as a cause of ESRD in Ireland. The 24 individuals currently living with ESRD included in this case series would alone account for 0.54% of ESRD in Ireland [15]. Although we sought to identify all cases of ADTKD by identifying individuals and their relatives through the IKGP study and by seeking referrals to the national renal genetics clinic this may still be an underestimate due to incomplete case ascertainment. Variation between estimates of prevalence may reflect regional or ascertainment differences.

A key recommendation of the KDIGO 2015 consensus report was the establishment of registries of patients with ADTKD to facilitate further research [7]. We have created a database of such patients in Ireland. In the future, increased awareness of the condition may lead to further individuals being identified.

This study further emphasizes the complexity in genetic testing for ADTKD. In 2/5 (40%) families with ADTKD-*MUC1* diagnosis was possible only due to the detection of MUC1fs protein in urinary cell smears.

The clinical characteristics of this group are similar to other cohorts [2,5,6]. In other reports, age at ESRD is variable, but typically occurs in middle age. The mean ages of ESRD for ADTKD-*MUC1*, ADTKD-*UMOD*, ADTKD-*HNF1B*, and ADTKD-NOS in this Irish cohort were 44, 47, 47, and 68 years, respectively. These differences were not statistically significant. Thirty-seven percent (20/54) of these patients had histological evidence of ADTKD. As expected, gout was a frequent comorbidity in the ADTKD-*UMOD* group (15/19, 78%).

Age at presentation was not significantly different between groups although ADTKD-NOS patients presented with higher creatinine and ADTKD-*MUC1* patients presented with lower creatinine. No patients in the ADTKD-*MUC1* group presented with ESRD at diagnosis. These differences may be, in part, due to earlier screening of family members in a particularly large ADTKD-*MUC1* family and demonstrates the value of accurate diagnosis of the disease in facilitating family-based screening.

In 4/16 (25%) families, we did not identify a pathogenic mutation in the genes known to cause ADTKD. This is not unique to our cohort – one Italian centre performed genetic testing in 21 families with suspected ADTKD and found a cytosine duplication in the VNTR of the *MUC1* gene in only 10% of cases [12]. Possible reasons for failure to identify a causative mutation include the complexity of sequencing and identifying mutations in the *MUC1* gene and existence of yet unrecognized causative gene mutations. Identification of MUC1fs protein in urinary smears is an important diagnostic tool in reducing cases of ADTKD without an established genetic diagnosis. In one of the still unsolved families we were unable to obtain fresh urine samples for immunohistochemical analysis for MUC1fs protein.

Awareness of ADTKD as a cause of CKD and ESRD is important for several reasons. Accurate diagnosis allows patients with CKD to be given prognostic information and planning for progression to ESRD [33]. Genetic diagnosis may allow transplantation from living related donors by determining that a potential donor is not themselves affected [34]. Greater awareness of ADTKD among clinicians will lead to better case ascertainment which allow for more accurate estimation of disease prevalence.

Establishing a renal genetics clinic led to diagnosis of ADTKD in twelve individuals from four families (Figure 1). This clinic was recently established and continues to receive referrals for individuals with a suspected genetic cause of ADTKD. Centralizing genetic referrals allows for nationwide access to testing for rare genetic variants. This testing has formerly been largely done outside Ireland, in collaboration with the Broad Institute, Cambridge, Massachusetts, and Wake Forest School of Medicine, Winston-Salem, North Carolina, and the First Faculty of Medicine, Charles University, Prague, Czech Republic.

In a research lab, we have recently tested individuals with suspected ADTKD using a gene panel testing approach. Continuing to resource the renal genetics clinic and strengthening national capacity to test for genetic causes of kidney disease are important measures to ensure Irish patients with kidney disease receive accurate, timely genetic diagnoses.

There are a number of limitations to this study. The IKGP did not include the whole Irish population. While we sought to identify other families with ADTKD through referrals through the renal genetics clinic and liaison with other nephrology units it is possible that other individuals and families with CKD due to ADTKD exist. ADTKD may therefore be considered to account for a minimum of 0.54% of cases of ESRD, actual prevalence may be higher. Genetic testing for ADTKD continues to evolve and it is possible that this will allow identification of other individuals and families in the future. Finally, this study may not be generalizable to other geographic regions where the prevalence of ADTKD, and underlying genetic mutations, are likely to be different.

In summary, we describe an Irish cohort of patients with ADTKD. Characteristics in our cohort are similar to previously reported data. This report highlights the need to consider a diagnosis of ADTKD in individuals with unspecified tubulointerstitial disease or a family history of kidney disease. This work has established an Irish database of patients with ADTKD who could be included in future work to identify novel mutations or in clinical trials of disease modifying agents.

## Supplementary Material

Supplemental Material
