# Supplementary material for: Autosomal dominant tubulointerstitial kidney disease (ADTKD) in Ireland
Source: Ren Fail. 2019 Sep 11;41(1):832–41. doi: 10.1080/0886022X.2019.1655452 (PMC6746258; doi:10.1080/0886022X.2019.1655452)
Supplement: Supplemental Material [file IRNF_A_1655452_SM2692.pdf]

ARL13B	CLCNKB	IQCB1	SLC3A1	MAGI2	SLC22A12
ACE	CLDN16	KAL1	SLC4A1	SGPL1	SLC2A2
ACTN4	CLDN19	KCNJ1	SLC4A4	CFH	SLC2A9
ADCK4	CNNM2	KIF7	SLC5A2	CFI	SLC7A7
AGT	COL4A1	KLHL3	SLC7A9	CFHR5	SLC9A3R1
AGTR1	COL4A3	LAMB2	SMARCAL1	CD46	TCTN3
AGXT	COL4A4	LMX1B	SOX17	C3	THBD
AHI1	COL4A5	LYZ	TCTN1	COL4A6	TMEM231
ANLN	COQ2	MKKS	TCTN2	GANAB	TSC1
APOA1	CRB2	MKS1	TMEM138	DZIP1L	VIPAS39
APOL1	CSPP1	MYH9	TMEM216	VHL	VPS33B
APRT	CTNS	MYO1E	TMEM237	ADAMTS13	WDPCP
AQP2	CUL3	NEK8	TMEM67	ANKS6	ZMYND10
ARHGAP24	DSTYK	NOTCH2	TNXB	APOE	ZNF423
ARHGDIA	EHHADH	NPHP1	TRIM32	BBS9	BICC1
ARL6	EYA1	NPHP3	TRPC6	C1QA	SIX1
ATP6V0A4	FAH	NPHP4	TTC21B	C1QB	SCARB2
ATP6V1B1	FGF20	NPHS1	TTC8	C1QC	CFD
ATP7B	FGF23	NPHS2	UMOD	C5orf42	CFHR2
AVPR2	FN1	NR3C2	WDR19	CA2	APOA2
B2M	FRAS1	OCRL	WDR35	CEP83	IFNG
B9D1	FREM2	OFD1	WDR73	CD151	TSC2
B9D2	FXYD2	PAX2	WNK4	CEP164	CCND1
BBS1	GATA3	PDSS2	WNT4	CFB	DCDC2
BBS10	GLA	PKD1	WT1	CFHR1	MAPKBP1
BBS12	GLI3	PKD2	XPNPEP3	CFHR3	SEC61A1
BBS2	GLIS2	PKHD1	MUC1	ETFA	JAG1
BBS4	GPC3	PLCE1	TBX18	ETFB	
BBS5	GRHPR	PTPRO	CHRM3	ETFDH	
BBS7	GRIP1	REN	FREM1	FAN1	
BMP4	HNF1B	RET	HPSE2	GSN	
BSND	HNF4A	ROBO2	ITGA8	LCAT	
CASR	HOGA1	RPGRIP1L	LRIG2	MASP1	
CC2D2A	HSD11B2	SALL1	COQ6	MEFV	
CD2AP	IFT122	SCNN1B	DGKE	PKHD1L1	
CEP290	IFT140	SCNN1G	EMP2	PRPS1	
CEP41	IFT43	SIX5	ITGA3	RRM2B	
CHD1L	INF2	SLC12A1	NUP93	SARS2	
CLCN5	INPP5E	SLC12A3	NUP107	SDCCAG8	
CLCNKA	INVS	SLC34A1	NUP205	SLC1A1	

Supplementary Table 1: List of genes included in gene panel
